# In Silico Drug Repurposing of FDA-Approved Drugs Highlighting Promacta as a Potential Inhibitor of H7N9 Influenza Virus

**DOI:** 10.3390/molecules27144515

**Published:** 2022-07-15

**Authors:** Sphamandla E. Mtambo, Hezekiel M. Kumalo

**Affiliations:** Drug Research and Innovation Unit, Discipline of Medical Biochemistry, School of Laboratory Medicine and Medical Science, University of KwaZulu-Natal, Durban 4000, South Africa; sphamtambo@gmail.com

**Keywords:** virtual screening, drug repurposing, in silico method, molecular dynamics simulations, influenza A virus, H7N9, FDA-approved drugs

## Abstract

Influenza virus infections continue to be a significant and recurrent public health problem. Although vaccine efficacy varies, regular immunisation is the most effective method for suppressing the influenza virus. Antiviral drugs are available for influenza, although two of the four FDA-approved antiviral treatments have resulted in significant drug resistance. Therefore, new treatments are being sought to reduce the burden of flu-related illness. The time-consuming development of treatments for new and re-emerging diseases such as influenza and the high failure rate are increasing concerns. In this context, we used an in silico-based drug repurposing method to repurpose FDA-approved drugs as potential therapies against the H7N9 virus. To find potential inhibitors, a total of 2568 drugs were screened. Promacta, tucatinib, and lurasidone were identified as promising hits in the DrugBank database. According to the calculations of MM-GBSA, tucatinib (−54.11 kcal/mol) and Promacta (−56.20 kcal/mol) occupied the active site of neuraminidase with a higher binding affinity than the standard drug peramivir (−49.09 kcal/mol). Molecular dynamics (MD) simulation studies showed that the C-α atom backbones of the complexes of tucatinib and Promacta neuraminidase were stable throughout the simulation period. According to ADME analysis, the hit compounds have a high gastrointestinal absorption (GI) and do not exhibit properties that allow them to cross the blood–brain barrier (BBB). According to the in silico toxicity prediction, Promacta is not cardiotoxic, while lurasidone and tucatinib show only weak inhibition. Therefore, we propose to test these compounds experimentally against the influenza H7N9 virus. The investigation and validation of these potential H7N9 inhibitors would be beneficial in order to bring these compounds into clinical settings.

## 1. Introduction

In March 2013, an avian influenza A (H7N9) virus infection was discovered in humans in East China. Since then, it has infected 1565 people and killed approximately 39% of those who have been confirmed to have the Asian H7N9 virus infection [1,2]. Some influenza A (H7N9) virus-infected humans developed pneumonia and acute respiratory distress syndrome, with high case fatality rates [3,4]. The influenza A virus (IAV) has two major surface glycoproteins that dominate the virus surface: neuraminidase (NA) and hemagglutinin (HA). The cleavage of α-(2-3 or 2-6)-ketosidic linkage between terminal sialic acid and adjacent surface glycoprotein is catalysed by NA. Furthermore, this promotes the budding of newly formed viral particles from the infected cell, allowing progeny viruses to infect uninfected host cells and infect respiratory tract mucins [5,6].

HA is responsible for the attachment of the influenza virus to the infected host cell’s surface glycoprotein’s sialic acid. Avian virus HA proteins specifically recognize an α-2,3-linkage found on the epithelial cells of duck intestines. On the other hand, human virus HA proteins prefer sialic acid linked to galactose via an α-2,6-linkage expressed on the surface of human respiratory epithelial cells [7,8,9]. The influenza A (H7N9) virus has a higher affinity for the human α-2,6-linked sialic acid host cell receptor and a lower affinity for the avian α-2,3-linked sialic acid host cell receptor [10]. Serological studies have found no pre-existing immunity to H7 subtype influenza viruses present in humans [8,11]. As a result, the influenza A (H7N9) virus should be closely monitored due to its potential to cause a pandemic. Research on the development of more potent anti-influenza drugs should be prioritized.

NA and adamantanes drugs are the two traditional classes approved for the treatment of IAV infection [6,12]. NA inhibitors are the only way to treat influenza infection in most countries due to the mutation and subsequent resistance to adamantane drugs (amantadine and rimantadine) [13]. There are three Food and Drug Administration (FDA)-approved NA inhibitors for treating influenza infection: zanamivir, oseltamivir, and peramivir. At present, laninamivir is only licensed in Japan [2,14,15]. The FDA recently approved a polymerase inhibitor baloxavir marboxil in Japan and the United States as a possible antiviral option against IAV and IBV infection [16,17].

**Figure 1:**
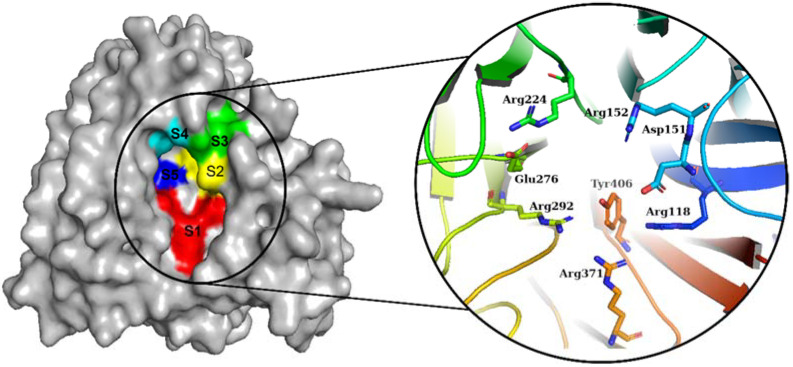
Diagram of the NA binding pocket showing five inhibitory binding subsites (S1 to S5) and conserved enzymatic residues.

The NA from IAVs is divided into two phylogenetic groups: group 1 (N1, N4, N5, and N8) and group 2 (N1, N4, N5, and N8) (N2, N3, N6, N7, and N9). NA is a homotetramer with an active site in each subunit [6,18]. In IAV and IBV, the active site forms a pocket composed of 19 highly conserved amino acid residues. The inner cavity contains eight highly conserved enzymatic residues (N2 numbering) that interact directly with the sialic acids responsible for enzymatic activity (Arg118, Asp151, Arg152, Arg224, Glu276, Arg292, Arg371, and Tyr406) (Figure 1). Furthermore, the rim contains 11 highly conserved framework residues that stabilize the enzymatic binding pocket (Glu119, Arg156, Trp178, Ser179, Asp (or Asn in N7 and N9) 198, Ile222, Glu227, His274, Glu277, Asp294, and Glu425) [5,19].

While vaccination remains one of the primary prevention strategies, it may not provide adequate protection in some seasons [6]. As such, preventive and therapeutic agents, and antiviral drugs are critical to the control of seasonal influenza epidemics as well as the ongoing fight against a pandemic outbreak. Consequently, it is critical that more novel medical therapies be identified as soon as possible for both preventive and therapeutic purposes. Developing novel therapeutic drugs, on the other hand, is a lengthy, time-consuming, and resource-intensive process that is plagued by more failures than successes [20].

In silico modelling is a useful approach for reducing the cost and time required for drug development [21]. The in silico technique has received a lot of attention as a tool for finding new drug leads, understanding disease mechanisms, and researching drug–target interactions [22,23]. As a result, it has been recognized as a valuable tool in balanced planning and the discovery of potential novel drugs [24]. Virtual screening has improved drug discovery and is now one of the most promising in silico approaches for drug design and development [24].

Existing, licensed drugs could be repurposed to produce more therapeutic drugs faster. When compared to the process of generating a drug from scratch, drug repurposing has the potential to significantly reduce development time and cost. This is due to the availability of toxicity and safety data from previous clinical trial phases. In the current study, we used molecular docking studies to perform in silico-based repurposing of FDA-approved drugs against influenza A (H7N9). Peramivir was utilized as a control drug in the comparative investigations. The stability of hit compounds complexed with viral NA was investigated using molecular dynamics (MD) simulations. In addition, in silico approaches were employed to predict the pharmacokinetic and toxicological properties of the hit compounds.

## 2. Results and Discussion

### 2.1. Molecular Docking Analysis

The DockRMSD server v1.1 was used to evaluate the accuracy of AutoDock Vina v.1.1.2 docking for higher hit rates in our virtual screening. The RMSD (without considering hydrogen atoms) value of co-crystal bound peramivir and re-docked peramivir was found to be 1.78 Å, indicating the great reliability of the docking process [25]. The interaction profiles of the three highest-scoring docking poses were examined and compared to the peramivir binding profile (Table 1). The binding affinities of the hit compounds were found to be greater than those of the peramivir compound (−6.8 kcal/mol), with Promacta having the greatest binding affinity (−10 kcal/mol), followed by lurasidone (−9.9 kcal/mol), and finally tucatinib (9.8 kcal/mol). The top 20 highest-scoring docking poses have been provided in Table S1 of the Supplementary Material.

### 2.2. Binding Pose Analysis

**Figure 2:**
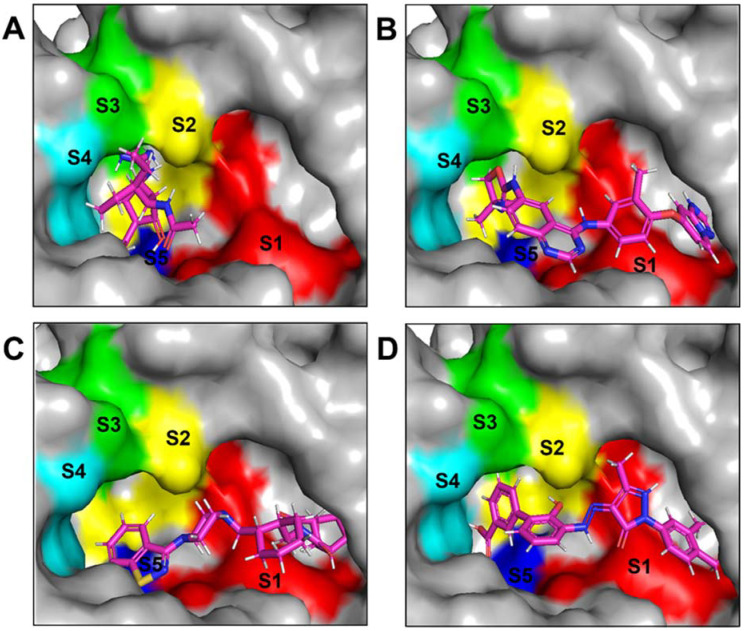
Binding modes of NA in complex with peramivir (**A**), lurasidone (**B**), tucatinib (**C**), and Promacta (**D**).

The NA binding site is made up of five subsites: S1, S2, S3, S4, and S5. The S1 site is made up of three positively charged arginine residues: Arg118, Arg292, and Arg371. The S2 site is a positively charged region composed of Glu119 and Glu227 residues, whereas the S3 site is a small hydrophobic region composed of Trp178 and Ile222 residues. The S4 site is a hydrophobic area that includes the residues Ile222, Arg224, and Ala246. Site S5 is a mixed polarity area composed of Glu276 and Ala246 residues. Our studies have demonstrated that Promacta, tucatinib, and lurasidone interact with the NA protein and share the same binding pocket, with interaction profiles comparable to that of peramivir’s binding pattern (Figure 2).

**Figure 3:**
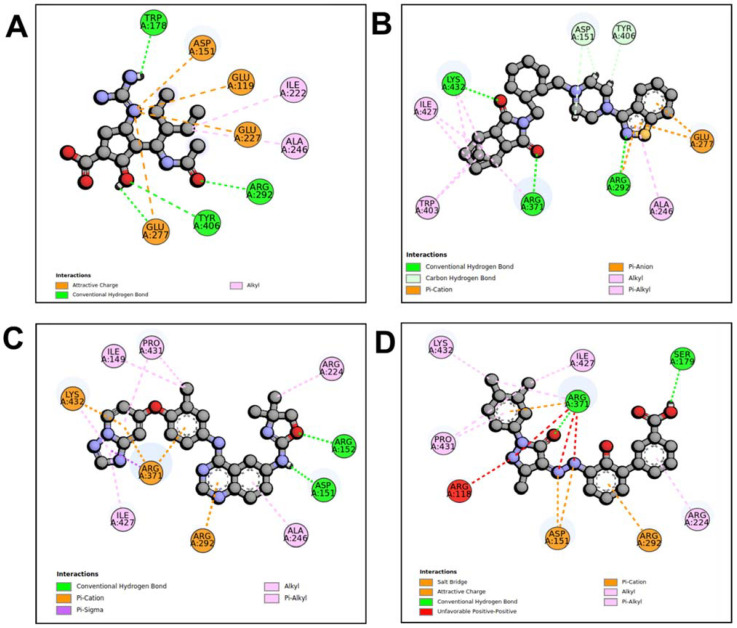
Molecular interaction profiles of NA with peramivir (**A**), lurasidone (**B**), tucatinib (**C**), and Promacta (**D**).

When compared to other hit compounds, peramivir indicated the least favourable binding affinity of −6.8 kcal/mol. According to our findings, peramivir occupies the binding site and interacts with residues through hydrogen bonds and hydrophobic interactions. Our results suggest that peramivir accommodates in the S1, S2, S3, and S4 binding subsites (Figure 2A). Peramivir established hydrogen bonds with Trp178, Arg292, and Tyr406 in the framework amino acids Glu119, Asp151, Trp178, Ile222, Arg227, Glu227 Ala246, Glu277, Arg292, and Tyr406 (Figure 3A).

Lurasidone appears to accommodate more in the S1 and S2 subsites of the binding pocket (Figure 2B). Lurasidone also interacted with enzymatic amino acid residues in the active site, including Asp151, Arg292, Arg371, and Tyr406, and framework residues such as Glu277. As seen in Figure 3B, the lurasidone compound formed hydrogen bonds with Arg292, Arg371, and Lys342 amino acids, showing potential good inhibitory activity (Figure 3B).

Tucatinib exhibited a binding energy of −9.8 kcal/mol, which is more favourable when compared to peramivir (−6.8 kcal/mol). Our study showed that tucatinib interacts with Ile149, Asp151, Arg152, Arg224, Ala246, Arg292, Asp294, Arg371, Ile427, Lys432, and Pro431, where it forms hydrogen bonds with three of the active site residues, Asp151, Arg152, and Asp294 (Figure 3C). Tucatinib also showed interactions with enzymatic amino acid residues Asp151, Arg152, Arg224, Arg292, and Arg371, and a structural framework residue Asp294. These interactions appear throughout all the subsites (S1, S2, S3, S4, and S5) located within the NA binding pocket (Figure 2C).

Promacta showed the most favourable binding energy of −10.0 kcal/mol in this study. It was found that Promacta forms an interaction with conserved enzymatic residues such as Arg118, Asp151, Arg224, Arg292, and Arg 371 (Figure 3D). Along with these residues, interaction with the framework residue Ser179 was also found. Additionally, Promacta formed hydrogen bonds with Arg371 and Ser179. Promacta appears to accommodate more in the binding pocket’s S1, S2, and S4 subsites (Figure 2D). The interaction with these residues is the most critical determinant in the orientation and stability of the NA complex with Promacta.

### 2.3. Molecular Dynamics Trajectory Analysis

To further evaluate the potential ability of lurasidone, tucatinib, and Promacta to act as an efficient inhibitor of NA, MD simulations were performed. In addition, MD analyses were performed to analyse and compare the dynamic behaviours of NA complexes with lurasidone, tucatinib, Promacta, and the reference ligand peramivir.

#### 2.3.1. Root Mean Square Deviation (RMSD)

**Figure 4:**
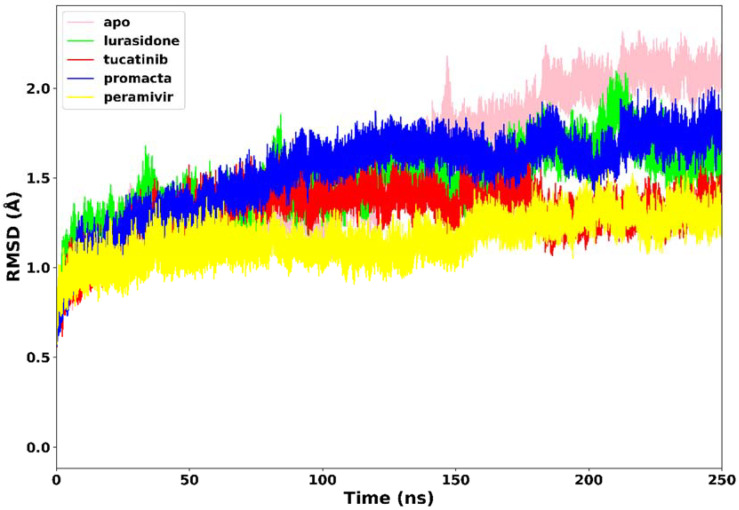
The RMSD trajectories of NA–ligand complexes during 250 ns simulations.

All four systems’ stability was investigated using RMSD of the C-α atoms. We analysed the structural stability of the docked compounds in the binding site and their effects on the overall system stability. During the simulations, the RMSD values of the entire system gradually converged and finally reached equilibrium at around 50 ns (Figure 4). The apo-enzyme showed an average RMSD value of 1.71 Å during the entire run. Lurasidone, tucatinib, and Promacta as well as the reference ligand, peramivir, complexed with NA exhibited average RMSD values of 1.48 Å, 1.31 Å, 1.53 Å, and 1.17 Å, respectively, which are lower than that of apo-enzyme. This shows that the presence of a ligand stabilizes the structure of the NA protein. The average RMSD for all the systems was found to be 1.40 Å, which is lower than the ideal 2 Å RMSD value [26]. According to the findings, all three systems were stable, which could be due to strong hydrogen bond interactions between the protein–ligand complexes.

#### 2.3.2. Root Mean Square Fluctuation (RMSF)

**Figure 5:**
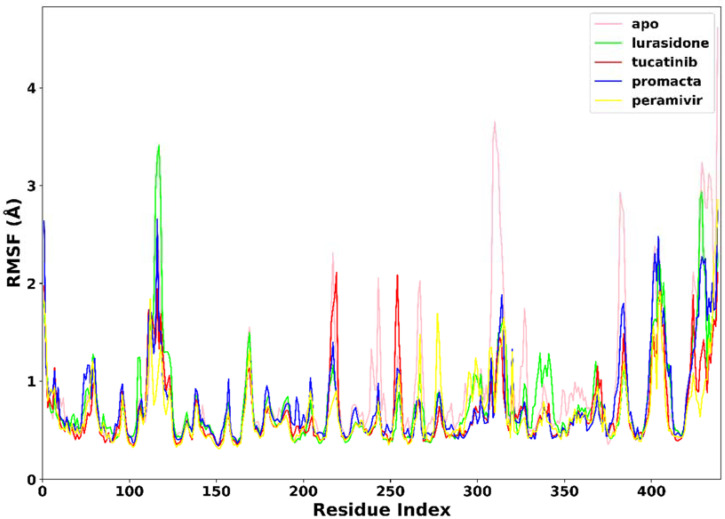
The RMSF trajectories of NA–ligand complexes during 250 ns simulations.

RMSF was used to measure the flexibility of the protein residues in terms of the C-α atom fluctuations throughout the MD simulation. As shown in Figure 5, the RMSF for individual amino acids correlates with the trend observed in the RMSD of complexes. The average RMSF was found to be 0.90 Å in the apo-enzyme, 0.78 Å in the lurasidone, 0.69 Å in the tucatinib, 0.67 Å in the Promacta, and 0.67 Å in the reference peramivir–NA complex. According to our findings, the presence of a ligand at the binding site reduces amino acid flexibility. In all systems, the pattern of residual flexibility is almost identical. Lower fluctuations were observed in the active site residues ranging from residues 110–120, 150–151, 152–160, 230–230, and 270–280, corresponding to the S1, S2, S3, S4, and S5 binding subsites, respectively. These findings show that the target protein is stabilized by binding all three compounds docked against it.

#### 2.3.3. Radius of Gyration (RoG)

**Figure 6:**
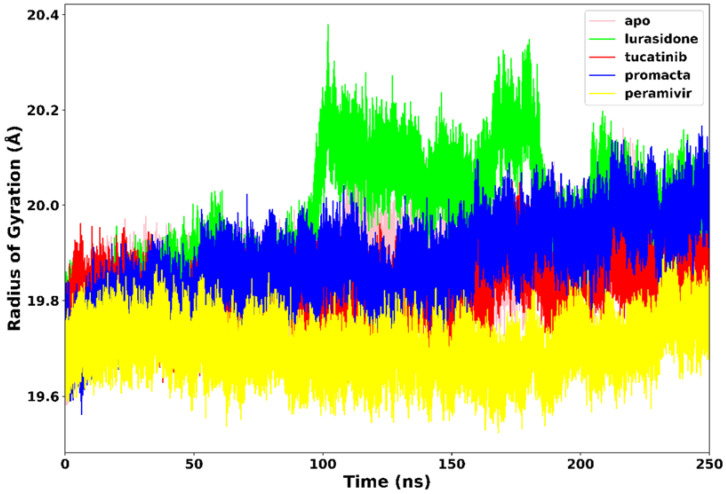
Radius of gyration trajectories of NA–ligand complexes during 250 ns simulations.

The RoG communicates information about the complex’s stable and unstable folding behaviour during the interaction of a protein and a ligand. A high RoG value indicates low structural compactness, whereas a low RoG value indicates high structural compactness [27]. RoG was therefore used to determine our system’s compactness during the simulation. Figure 6 demonstrates that the apo-enzyme had a higher average RoG value of 20.1 Å across the 250 ns simulation period than the complexes. Peramivir, lurasidone, tucatinib, and Promacta complexes exhibited average RoG values of 19.7 Å, 19.9 Å, 19.8 Å, and 19.8 Å, respectively. Contrary to lurasidone, the presence of peramivir, tucatinib, and Promacta at the NA active site appears to exert conformational stability and compactness. The NA–lurasidone complex’s higher fluctuations between 100 and 190 ns are assumed to be due to the binding and unbinding of lurasidone in the active site, thus affecting the overall compactness of the complex.

#### 2.3.4. Hydrogen Bond Analysis

**Figure 7:**
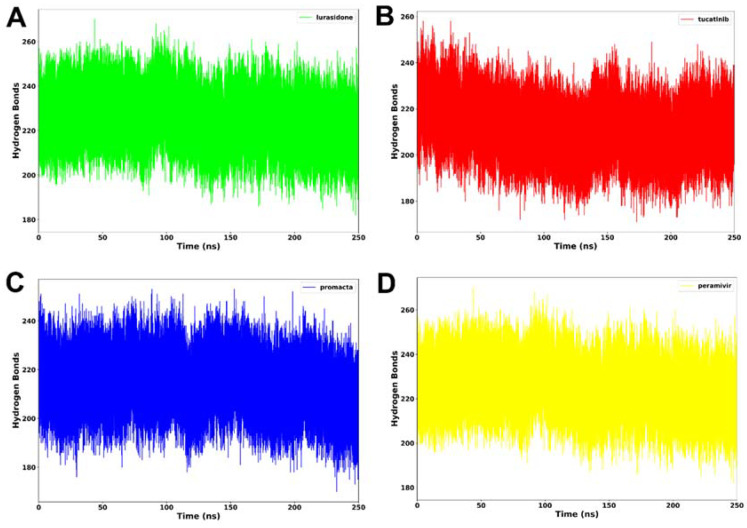
Hydrogen bond formation of NA complexed with lurasidone (**A**), tucatinib (**B**), Promacta (**C**), and peramivir (**D**) during 250 ns simulations.

Hydrogen bonding between residues is an important metric for determining a protein’s stability. A protein structure’s stability may be increased by the presence of more intermolecular hydrogen bonds. Interactions at the binding site, such as hydrogen bonds, hydrophobic interactions, and ionic interactions, largely determine ligand binding affinity [28]. An average of 212 hydrogen bonds was consistently formed in the NA–lurasidone complex (Figure 7). In the cases of the tucatinib– and Promacta–NA complexes, an average of 214 and 217 hydrogen bonds were consistently formed throughout the simulation period, respectively. Throughout the simulation, an average of 225 hydrogen bonds were formed with the reference ligand, peramivir. In comparison to lurasidone and tucatinib, our analysis revealed that Promacta formed more hydrogen bonds with other amino acids over the simulation time. This could be attributed to the NA–Promacta complex’s conformational stability, which could indicate a stronger binding affinity.

**Figure 8:**
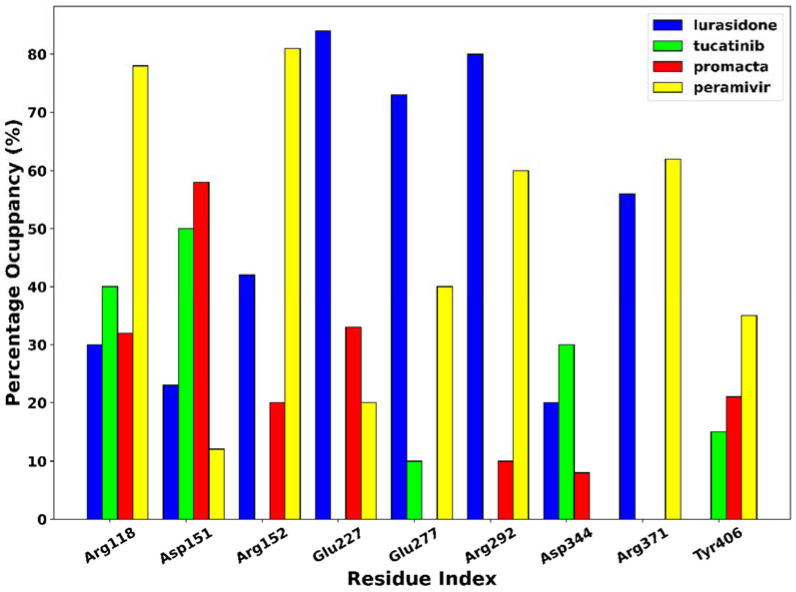
Hydrogen bond occupancy of NA–ligand complexes during 250 ns simulations.

The phenomenon of hydrogen bonding was studied to determine which residues at the bonding site contribute to hydrogen bonding. For this purpose, the percentage occupancy of the hydrogen bonds was investigated. Throughout the 250 ns simulation time, the percentage of hydrogen bond occupancy (%) between the ligands and the active site residues was monitored (Figure 8). Based on our results, amino acid residues Arg118, Asp151, Arg152, Glu227, Glu277, Arg292, Asp344, Arg371, and Try406 were identified as the primary residues for hydrogen bonding between NA and the hit compounds, with the highest percentage occupancy, suggesting a significant contribution to system stabilisation. The interaction of the ligands with these primary residues has a significant impact on the efficacy of the ligand. As shown in Figure 6, the complexes of peramivir–, Promacta–, lurasidone–, and tucatinib–NA formed hydrogen bonds with Arg118 at 78%, 40%, 30%, and 32% occupancy throughout the simulation runs. Furthermore, Arg118 and Asp151 were found in nearly all complexes.

### 2.4. Binding Free Energy Analysis

The binding free energy contributions for NA–ligand complexes over the simulation time are shown in Table 2.

Non-bond interactions, such as the van der Waal energy (ΔE_vdw_) and the electrostatic energy (ΔE_ele_), have a major impact on the estimation of the binding free energy (G_bind_) in energy calculations. According to the binding free energy calculations, the compounds Promacta and tucatinib have the highest binding affinity for NA with a ΔG_bind_ of −54.11 ± 0.11 and −56.20 ± 0.19 kcal/mol, respectively, compared to the reference compound peramivir (−49.09 ± 0.13 kcal/mol). Compared to peramivir, the higher ΔE_vdw_ energy plays an essential role in the ΔG_bind_ binding energies of tucatinib and Promacta. This is due to the presence of hydrophobic interactions between amino acid residues in the binding site, which contribute to the stabilisation of the conformation of the Promacta–NA complex. According to our findings, tucatinib– and Promacta–NA can effectively inhibit by binding to the active site and impeding catalytic activity. Therefore, we recommend that tucatinib and Promacta be investigated for their potential use in the treatment of influenza virus infections. Due to limitations associated with approximations in the binding free energy calculations, the binding free energy values represent a trend for NA–ligand complexes.

### 2.5. Interaction Energy Decomposition Analysis

**Figure 9:**
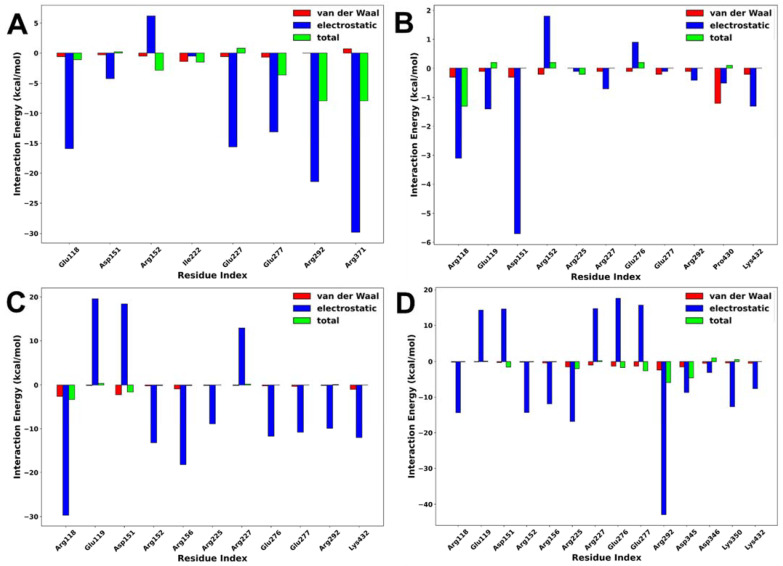
Interaction energy decomposition of NA complexed with peramivir (**A**), lurasidone (**B**), tucatinib (**C**), and Promacta (**D**).

Interaction energy decomposition analysis was performed to gain an insight into the contributions of individual amino acids to the total free energy of binding of protein–ligand complexes. As shown in Figure 9, the amino acids with the highest residual energy contributions were Arg118 (−3.12 kcal/mol) and Asp151 (−5.74 kcal/mol) in the NA–lurasidone complex, Arg118 (−29.74 kcal/mol), Arg152 (−13.21 kcal/mol), and Arg156 (−18.29 kcal/mol) in the NA–tucatinib complex, Arg118 (−14.47 kcal/mol), Arg225 (−16.83 kcal/mol), and Arg292 (−42.90 kcal/mol) in the NA–Promacta complex, and Arg119 (−15.91 kcal/mol), Arg292 (−24.47 kcal/mol), and Arg371 (−29.85 kcal/mol) in the reference compound peramivir.

The electrostatic interactions contributed much more to the total binding energy than the van der Waals interactions. These residues are thought to be important components of the protein–ligand binding pocket. Compared to the tucatinib and Promacta complexes, the NA–lurasidone complex contributed the least residue energy. The thermodynamic stability and protein–ligand solid interactions in the tucatinib– and Promacta–NA complexes are thought to be due to the overall higher electrostatic contribution and lower flexibility of the backbone C-α atoms. Furthermore, Arg118 was found in all complexes.

### 2.6. Pharmacokinetic Analyses

An assessment of how an active ingredient is absorbed into the body, distributed, metabolised into its many components, and excreted from the body is very important for optimising the active ingredient during drug discovery. This section evaluates the absorption, distribution, metabolism, and excretion (ADME) properties of hit compounds using an in silico Swiss-ADME server to understand pharmacokinetic properties.

Table 3 shows that, unlike peramivir, all hit compounds have high gastrointestinal absorption (GI). The ability of a drug to cross the blood–brain barrier (BBB) is a prerequisite for a drug to have effects on the central nervous system. However, if the effect of the drug is needed in other tissues, passage through the BBB can have unfavourable consequences [29]. In the case of hit substances, they lack properties that allow them to pass through the BBB and cause adverse effects.

Cellular efflux pumps such as P-glycoprotein (P-gp) act as a first line of protection by transporting toxic xenobiotics and toxic substances out of the cell [30]. It is a substrate for many structurally varied therapeutics, and it prevents drug absorption, permeability, and retention, and expels them out of the cells [31]. Notably, our analysis indicates that in contrast to tucatinib, lurasidone and Promacta are not P-gp substrates, suggesting the possibility of successful drug delivery. The drug metabolism of pharmaceuticals and other xenobiotics by cytochrome (CYP) enzymes produced variable results. All compounds demonstrated favourable ADME properties, indicating a high potential for their use as lead compounds.

### 2.7. Toxicological Analyses

In silico methodologies may now be used to determine the safety profiles of desired substances thanks to computerized technologies. These chemicals might damage humans and animals if they are not used properly. The server ProTox-II was used to analyse the safety profiles of our hit compounds (Table 4). According to the findings, tucatinib is the only compound with carcinogenic and immunotoxic adverse effects.

Furthermore, all compounds show negative results for mutagenicity and cytotoxicity prediction. According to the ProTox-II server, tucatinib and Promacta both belong to class 5, with LD_50_ ranging from 2000 to 5000 mg/kg, and may be harmful when administered orally. The LD_50_ value for lurasidone was less than 2000 mg/kg, indicating that oral ingestion could be harmful, putting it in toxicity class 4.

Inhibition of human ether-a-go-go-related gene (hERG) channels is a widely accepted predictor of cardiotoxicity. Screening compounds in the early stages of discovery and development for their ability to inhibit the hERG channel has thus become an essential procedure in the pharmaceutical industry. The Pred-hERG server was used to predict the ability of the hit compounds to inhibit hERG, and the results show that Promacta is non-cardiotoxic, while lurasidone and tucatinib only show weak inhibition.

## 3. Materials and Methods

### 3.1. Receptor and Ligand Preparation

The Protein Data Bank was used to obtain the X-ray crystal structure of the Anhui N9–peramivir (PDB code: 4MWV) complex. AutoDock Tools (ADT) v.1.5.6 was used to prepare the receptor for docking [32,33]. Water molecules, ions, and ligands were removed from the protein, which was then followed by the construction of missing side chain atoms, the addition of hydrogen atoms, and the protonation of individual residues. Following that, 1000 steps of receptor minimization were conducted with the conjugate gradient algorithm using MMFF94 force field, and the PDB file was translated to PDBPT file format using Open Babel v.2.4.1 [34].

A total of 2568 FDA-approved drugs available for virtual screening were obtained from DrugBank database [35]. They were then filtered using Open Babel to select only drugs that meet Lipinski rule of five [36], filtering only 4 descriptors: partition coefficient, molecular weight, hydrogen bond donors, and acceptors, yielding 1597 drugs for virtual screening. The co-crystal bound peramivir, was used as a reference compound. The SDF files were hydrogenated at pH 7.4, applied using 1000 steps of a conjugate gradient algorithm using an MMFF94 force field, and then converted to PDBPT format using Open Babel.

### 3.2. Molecular Docking-Based Virtual Screening

Molecular docking was carried out using AutoDock Vina v.1.1.2 [33]. ADT was used to calculate the grid, which includes the binding centre and dimensions. A grid box centred at x = 63.17, y = 16.81, and z = −26.38 with the side lengths of x, y, and z set, respectively, at 50.0, 50.0, and 50.0 was positioned in the centre of the receptor’s binding site. Prior to conducting virtual screening with the selected compounds, AutoDock Vina was validated for its ability to reproduce the crystallographic pose of co-crystallized peramivir with NA. The RMSD values of docked peramivir and co-crystal bound peramivir were calculated using the DockRMSD server v1.1 [25]. The docking data were evaluated and the top ten drug conformations with the highest scores were chosen for additional docking. Using an in-house bash script, these conformations were docked in triplicate to the binding site using 24 exhaustiveness. The top 3 docked compounds with the best binding affinities were visualized using Discovery Studio Visualizer version 20.1.0.19295 [37], and PyMol v.2.5 [38].

### 3.3. Pharmacokinetic and Toxicological Predictions

The Swiss-ADME server was utilized to collect information on the physicochemical and pharmacokinetic properties of the hit drugs in this study [39]. Assessment of toxicity was performed using the ProTox II server [40]. The Pred-hERG server was used to predict the capacity of the ligands to inhibit hERG (Kv11.1) [41].

### 3.4. Molecular Dynamics Simulations

MD simulations of 250 ns were run with the best-scored poses of the screening complexes Promacta, tucatinib, and lurasidone, as well as the reference ligand peramivir. Amber 18 graphic processing unit (GPU) Particle Mesh Ewald Molecular Dynamic (PMEMD) was employed in the MD simulations. The force field-related parameters and protein descriptions were handled with FF14SB [42,43]. LEAP module was used in the addition of hydrogen atoms to the protein and subsequent counter-ions addition [44]. The systems were enclosed in TIP3P water box, with a 10 Å distance between the system surface and box edge [45]. The system employed the periodic boundary conditions, while long range electrostatics was managed with 12 Å Van der Waals cut off. The initial minimization was performed using restrained potential of 500 kcal/mol/Å^2^ in 1000 steepest descent steps and 1000 conjugate gradient steps on the solute [46]. This was followed by 1000 steps unrestrained conjugate gradient minimization for the entire system. A gradual heating from 0 to 300 K with 1 ps, 5 kcal/mol/Å^2^ (collision frequency and harmonic restraints, respectively) settings using Langevin thermostat was applied to the system. An unrestrained equilibration of the system was performed using NPT ensemble at 300 K and 1 bar constant pressure [47]. MD simulation production run of 250 ns was performed using an isothermal isobaric (NPT) ensemble and a Berendsen barostat [48]. The coordinates were saved at intervals after each stage and the trajectories were analysed.

### 3.5. Molecular Dynamics Trajectory Analyses

The Amber18 implemented modules, PTRAJ and CPPTRAJ, were used to perform post-MD trajectory studies such as root mean square deviation (RMSD) and root mean square fluctuations (RMSF), radius of gyration (RoG), number of hydrogen bonds, and hydrogen bond occupancy. All plots were created with Python v3.10.0 custom scripts and the Pandas v1.4.1 and Matplotlib v3.5.0 libraries.

### 3.6. Binding Free Energy Calculation

The binding free energy profiles of the best docking poses of Promacta, tucatinib, and lurasidone, and also reference compound, peramivir, complexed with NA were computed using the Molecular Mechanics/Generalized Born Surface Area (MM/GBSA) approach [49,50,51,52]. The following equations provide a full description of how to calculate binding free energy:

ΔG_bind_ = G_complex_ − G_receptor_ − G_ligand_ (1)

ΔG_bind_ = E_gas_ + G_sol_ − TΔS (2)

E_gas_ = E_int_ + E_vdW_ + E_ele_ (3)

G_sol_ = G_GB_ + G_SA_ (4)

In this equation, ΔG_bind_ indicates total free binding energy, whereas others reveal the free energy of complex, the protein, and the ligand. The gas phase energy is denoted by E_gas_, while internal energy is denoted by E_int_. The temperature is represented by T, and the total solute entropy is represented by ΔS. Interactions between bonded, electrostatic, and van der Waals states are specified by G_bind,_ E_ele_, and E_vdw_, respectively. G_GB_ and G_SA_, on the other hand, represent the polar and non-polar interaction with free energy. The MM/GBSA method was also utilized to calculate the energy contributions of individual amino acid residues to the overall binding free energy.

## 4. Conclusions

The newly developed drugs showed binding in the active site of NA in a highly specific binding pattern similar to that of peramivir with this in silico approach. According to our findings, some of the selected compounds occupied the active site of NA with an even higher binding affinity than peramivir. The MM-GBSA calculations showed that lurasidone and Promacta had higher binding affinity than the standard drug peramivir (−49.09 kcal/mol), with ΔG_bind_ values of −54.11 kcal/mol and −56.20 kcal/mol, respectively. The MD simulation studies revealed that the backbone of C-α atoms in the complexes of tucatinib and Promacta NA is stable throughout the simulation period and is not subject to significant fluctuations. In contrast, RoG analysis of the NA–lurasidone complex revealed higher fluctuations between 100 and 190 ns, affecting the complex’s stability. The major amino acid residues with the highest energy contributions were identified, providing a solid basis for future research into novel and effective influenza virus inhibitors. According to ADME analysis, the hit compounds have a high GI and do not possess properties that allow them to overcome the BBB. In silico toxicity prediction revealed that all compounds tested negative for mutagenicity and cytotoxicity. In addition, Promacta is not cardiotoxic, while lurasidone and tucatinib have only weak inhibition. Promacta and tucatinib could be used as lead compounds to combat the influenza A (H7N9) virus.

## Figures and Tables

**Table 1:** Virtual screening results of the hit compounds.

DrugBank ID	Generic Name	Physicochemical Properties	Structures	Binding Residues	Binding Affinity (kcal/mol)	Function
DB06614	Peramivir	Mw = 328.41 logP = 0.08 HBA = 5 HBD = 6	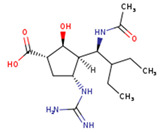	Glu119, Asp151, Trp178, Ile222, Arg227, Glu227 Ala246, Glu277, Arg292, Tyr406	−6.8	Treatment of influenza
DB08815	Lurasidone	Mw = 492.68 logP = 4.22 HBA = 4 HBD = 0	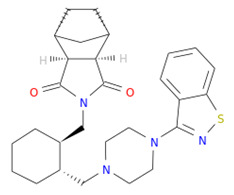	Asp151, Ala246, Glu277, Arg292, Arg371, Trp403, Tyr406, Ile427, Ly432	−9.9	Treatment of schizophrenia
DB11652	Tucatinib	Mw = 480.53 logP = 3.77 HBA = 7 HBD = 2	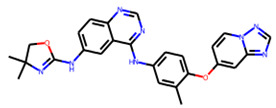	Ile149, Asp151, Arg152, Arg224, Ala246, Arg292, Asp294, Arg371, Ile427, Lys432, Pro431	−9.8	Treatment of metastatic breast cancer
DB06210	Promacta	Mw = 442.47 logP = 3.74 HBA = 6 HBD = 3	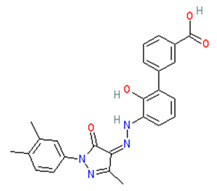	Arg118, Asp151, Ser179, Arg224, Arg292, Arg371, Ile427, Pro431, Lys432	−10.0	Treatment of thrombocytopenia or aplastic anaemia

logP—partition coefficient; Mw—molecular weight; HBD—hydrogen bond donors; HBA—hydrogen bond acceptors.

**Table 2:** Binding free energy contributions for NA–ligand complexes.

Complexes	ΔG_bind_	ΔE_vdw_	ΔE_ele_	ΔG_pol_	ΔG_nonpol_
NA–lurasidone	−22.59 ± 0.14	−28.20 ± 0.09	−32.20 ± 0.51	41.27 ± 0.44	−3.33 ± 0.01
NA–tucatinib	−54.11 ± 0.11	−57.95 ± 0.09	−41.76 ± 0.25	51.50 ± 0.22	−5.91 ± 0.02
NA–Promacta	−56.20 ± 0.19	−39.17 ± 0.12	−76.47 ± 0.43	65.07 ± 0.30	−5.66 ± 0.01
NA–peramivir	−49.09 ± 0.13	−28.86 ± 0.08	−128.21 ± 0.35	115.11 ± 0.26	−15.12 ± 0.00

ΔG_bind_—binding free energy; ΔE_ele_—electrostatic interaction; ΔE_vdw_—van der Waals forces; ΔG_pol_—polar salvation energy; ΔG_nonpol_—non-polar salvation energy.

**Table 3:** Comparative pharmacokinetics analyses.

Parameters	Lurasidone	Tucatinib	Promacta	Peramivir
GI absorption	High	High	High	Low
BBB permeant	No	No	No	No
P-gp substrate	No	Yes	No	Yes
CYP1A2 inhibitor	No	Yes	No	No
CYP2C19 inhibitor	Yes	Yes	No	No
CYP2C9 inhibitor	Yes	Yes	Yes	No
CYP2D6 inhibitor	No	Yes	No	No
CYP3A4 inhibitor	Yes	Yes	No	No

**Table 4:** Comparative toxicological analyses.

Parameters	Lurasidone	Tucatinib	Promacta	Peramivir
Carcinogenicity	No	Yes	No	No
Immunotoxicity	No	Yes	No	No
Mutagenicity	No	No	No	No
Cytotoxicity	No	No	No	No
LD_50_ (mg/kg)	660	3160	5000	1430
Class	4	5	5	4
hERG inhibition	Yes (weak)	Yes (weak)	No	No

## Data Availability

The data presented in this study are available on request from the corresponding author.

## Supplementary Materials

The following supporting information can be downloaded at: https://www.mdpi.com/article/10.3390/molecules27144515/s1, Table S1. Virtual screening results of the top 20 hit compounds.

## Sample Availability

Sample of the compounds are not available from authors.
