# Supplementary material for: In Silico Drug Repurposing of FDA-Approved Drugs Highlighting Promacta as a Potential Inhibitor of H7N9 Influenza Virus
Source: Molecules. 2022 Jul 15;27(14):4515. doi: 10.3390/molecules27144515 (PMC9321947; doi:10.3390/molecules27144515)
Supplement: Supplementary file 1 [file molecules-27-04515-s001.zip › molecules-1757490-supplementary.pdf]

Table S1. Virtual screening results of the top 20 hit compounds.

DrugBank ID	Generic Name	Binding Affinity (kcal/mol)
DB06210	Promacta	-10.0
DB08815	Lurasidone	-9.9
DB11652	Tucatinib	-9.8
DB11791	Capmatinib	-9.6
DB09280	Lumacaftor	-9.4
DB11703	Acalabrutinib	-9.3
DB15233	Avapritinib	-9.2
DB09074	Olaparib	-9.1
DB09383	Meprednisone	-9.1
DB13874	Idhifa	-9.0
DB15305	Risdiplam	-9.0
DB00619	Imatinib	-9.0
DB01260	Desonide	-9.0
DB14895	Vibegron	-8.9
DB00991	Oxaprozin	-8.9
DB01267	Paliperidone	-8.9
DB00293	Raltitrexed	-8.8
DB11760	Talazoparib	-8.8
DB11901	Apalutamide	-8.8
DB11942	Selinexor	-8.8
